# Evaluation of HER-2/neu gene amplification and protein expression in non-small cell lung carcinomas

**DOI:** 10.1038/sj.bjc.6600286

**Published:** 2002-05-06

**Authors:** F R Hirsch, M Varella-Garcia, W A Franklin, R Veve, L Chen, B Helfrich, C Zeng, A Baron, P A Bunn

**Affiliations:** Department of Pathology, Campus Box B216, University of Colorado Health Sciences Center, 4200 East 9th Avenue, Denver, Colorado, CO 80262, USA; Department of Medicine, Division of Medical Oncology, Campus Box B188, University of Colorado Health Sciences Center, 4200 East 9th Avenue, Denver, Colorado, CO 80262, USA; Department of Preventive Medicine and Biometrics, Campus Box B119, University of Colorado Health Sciences Center, 4200 East 9th Avenue, Denver, Colorado, CO 80262, USA; Tobacco Related Malignancy Program, University of Colorado Cancer Center, Campus Box B188, 4200 East 9th Avenue, Denver, Colorado, CO 80262, USA

**Keywords:** lung cancer, HER-2/neu, oncogene overexpression, immunohistochemistry, FISH, gene amplification

## Abstract

HER-2/neu gene amplification and cell surface overexpression are important factors in breast cancer for prognosis and prediction of sensitivity to anti-HER-2/neu monoclonal antibody therapy. In lung cancer, the clinical significance of HER-2/neu expression is currently under evaluation. We investigated 238 non-small lung carcinomas for HER-2/neu protein overexpression by immunohistochemistry using the HercepTest. We found 2+ or 3+ overexpression in 39 patients (16%), including 35% in adenocarcinomas and 20% in large cell carcinomas, but only 1% of squamous cell carcinomas. Marked (3+) overexpression was uncommon (4%). The association between protein expression and gene copy number per cell, as determined by fluorescence *in situ* hybridisation assay, was investigated in 51 of these NSCLC tumours. Twenty-seven tumours (53%) were negative by both tests. Marked (3+) protein expression and gene amplification were present in only 4% of samples. In 11 tumours (21%), gene gain was accompanied by chromosomal aneusomy and did not result in high protein levels while in 7 (14%) the score 2+ was associated with maximum number of signals per cell <9. The prognostic implication of HER-2/neu protein expression was studied in 187 surgically resected tumours. No statistical difference in survival was observed comparing patients with positive (2+/3+) and negative tumours (0/1+), although 3+ patients showed a tendency to shorter survival. The therapeutic implications of protein expression and gene amplification in lung cancer need to be examined in prospective clinical trials.

*British Journal of Cancer* (2002) **86**, 1449–1456. DOI: 10.1038/sj/bjc/6600286 www.bjcancer.com

© 2002 Cancer Research UK

## 

Lung cancer is the leading cause of cancer death in both men and women in the US (Greenlee *et al*, 2001). A better understanding of the biologic pathways that drive neoplastic cellular proliferation has led to the development of targeted therapies aimed at specific proteins in these pathways. Among these are trastuzumab (Herceptin^R^), a humanised monoclonal antibody that recognises the HER-2/neu protein receptor. The human HER-2/neu oncogene is located on chromosome 17 and encodes a transmembrane glycoprotein of 185 kD, which has intrinsic tyrosine kinase activity (Stern *et al*, 1986), and shares sequence homology with the epidermal growth factor receptor (Coussens *et al*, 1985; Slamon and Clark, 1988).

HER-2/neu overexpression has been observed by various diagnostic modalities in 20–30% of human breast cancers and is associated with a poorer overall survival and resistance to hormone based therapy (Slamon *et al*, 1987, 1989; Berchuck *et al*, 1990; Liu *et al*, 1992; Press *et al*, 1994; Pauletti *et al*, 1996; Allred and Swanson, 2000; Jacobs *et al*, 2000). Trastuzumab was approved by the US Food and Drug Administration (FDA) for the treatment of breast cancer patients whose tumours overexpress HER-2/neu. Trastuzumab produced objective responses in 26% of previously untreated breast cancer patients (Vogel *et al*, 2001) and 15% of breast cancer patients in the second line setting (Cobleigh *et al*, 1999). Response rates were considerably higher when trastuzumab was combined with doxorubicin or paclitaxel (45%), and breast cancer patients treated with the combination had higher response rates and longer survival than patients treated with doxorubicin or paclitaxel alone (Slamon *et al*, 2001).

Several studies evaluated HER-2/neu oncogene and protein expression in lung cancers using immunohistochemical (IHC) techniques (Schneider *et al*, 1989; Kern *et al*, 1990; Tateishi *et al*, 1991; Paakko *et al*, 1992; Tsai *et al*, 1993; Hsieh *et al*, 1998). These studies reported overexpression of HER-2/neu in 27–57% of patients with non-small cell lung carcinomas (NSCLC), with adenocarcinomas having the highest rates of overexpression. This wide variation in frequency is likely to be related to differences in the methodologies tested and patient populations studied. The overexpression of HER-2/neu in NSCLC has been associated with a poorer survival and shortened time to relapse (Tateishi *et al*, 1991; Hsieh *et al*, 1998; Brabender *et al*, 2001; Korrapati *et al*, 2001) but these studies were limited by small sample sizes and multivariate analysis was not performed. Additionally, in NSCLC cell lines, overexpression of HER-2/neu has been reported to be associated with resistance to chemotherapeutic agents (Tsai *et al*, 1993) and a synergistic effect was obtained when trastuzumab was combined with chemotherapeutic agents (Bunn *et al*, 2001). The application of trastuzumab for the treatment of human lung cancers that overexpress HER-2/neu is under investigation. In breast cancer, a comparison between IHC and fluorescence *in situ* hybridisation (FISH) analyses showed FISH to be the method of choice for predicting the clinical outcome (Pauletti *et al*, 2000). The method of choice and the level of HER-2/neu protein expression required to obtain a potential therapeutic effect from trastuzumab monoclonal antibody therapy has not yet been established in lung cancer.

The purpose of this investigation was to characterise HER-2/neu in primary lung cancers at protein and gene levels using the HercepTest and DNA FISH probes. Furthermore, the prognostic impact of the HER-2/neu protein expression was studied on a series of 187 tissue microarrayed lung tumours.

## MATERIALS AND METHODS

### Comparison of immunohistochemistry (IHC) and fluorescence *in situ* hybridisation (FISH)

For the comparison of IHC and FISH, we studied 51 patients with primary NSCLC treated at the University of Colorado and the Veterans Administration Hospitals, Denver, CO. Sections of tumour tissue were obtained by informed consent through the Histopathology Core of the University of Colorado Cancer Center. The tumour blocks were fixed in 10% buffered formalin for 5–10 h and embedded in paraffin. The blocks were cut in consecutive 4-micron sections. One section was stained with Hematoxylin and Eosin (H&E), and classified according to the World Health Organisation (WHO) histologic classification of lung tumours (Travis *et al*, 1999). Areas rich in viable tumour cells were selected in the H&E slides and subsequent sections were submitted for FISH and HercepTest analyses in a blinded fashion.

The clinical characteristics of these patients were as follows: There were 39 males and 12 females, and the median age was 65 years (range 46–79). According to the clinical stage, 15 patients were classified in stage 1A, 17 in stage IB, four in stage IIA, four in stage IIB and 11 in stage IIIA. Histologically, there were 24 adenocarcinomas, five large cell carcinomas (LCC), and 22 squamous cell carcinomas (SCC). As positive controls, we used 12 pre-selected breast cancer specimens taken from the Histopathologic Core and known to be strongly positive (3+) by the HercepTest method. In addition, 12 normal lung samples were selected from autopsy material from patients without any history of neoplastic or non-neoplastic pulmonary disease.

### Immunohistochemistry (IHC) assay

The IHC procedure followed the DAKO's protocol (DAKO Corporation, Glostrup, Denmark) for the HercepTest. In brief, the sections were deparaffinised, hydrated, and the antigen retrieval was performed at 95°C in citrate buffer for 40 min. The slides were then cooled at room temperature for 20 min, washed with TRIS-buffer 3×3 min, and the peroxidase blocking was performed for 5 min. After rewashing, the primary antibody was applied for 30 min. Following the application of the secondary antibody the substrate–chromogen solution (DAB) was added as a visualisation reagent. Finally the slides were counterstained with Hematoxylin.

Evaluation of the HercepTest followed the manufacturer's recommendation with a slight modification. Each sample was placed into one of four categories (0, 1+, 2+, 3+). Tumours with complete absence of staining were scored as 0, those with weak, incomplete membranous staining were classified as 1+. Tumours with either strong, incomplete basolateral staining or weak, complete membranous staining in greater than 10% of the tumour cells were classified as 2+, and those with strong, complete membranous staining in greater than 10% of the tumour cells were classified as 3+. According to the HercepTest protocol, all tumours classified as 2+ and 3+ were considered ‘positive’, and all tumours scored as 0 or 1+ were classified as ‘negative’. Two observers (WA Franklin and FR Hirsch) scored each slide, discrepancies were discussed, and a consensus score provided.

### Fluorescence *in situ* hybridisation (FISH) assay

Tissue sections were incubated at 65°C for 4 h, deparaffinized in three xylene washes for 10 min, and dehydrated in 100% ethanol. After incubation in 2×standard sodium citrate-SSC (pH 7.0) at 75°C for 20–25 min, sections were digested with proteinase K (0.25 mg ml^−1^ in 2×SSC, pH 7.0) at 37°C for 20–25 min, rinsed in 2×SSC (pH 7.0) at room temperature for 5 min, and dehydrated in ethanol series. Dual-target, dual-colour FISH assays were performed using the PathVysion Her2 DNA probe kit (Vysis, Downers Grove, IL, USA), including the LSI Her2 sequence labelled in SpectrumOrange and the chromosome 17 centromere sequence labelled in SpectrumGreen. The probe set was applied to the selected area on each slide, the hybridisation area was covered with a glass coverslip and sealed with rubber cement. The slides were incubated at 80°C for 10 min for co-denaturation of chromosomal and probe DNA's and hybridisation was allowed to occur in a humidified chamber at 37°C for 16–20 h. Post-hybridisation washes were performed in 1.5 M urea/0.1×SSC (pH 7.0–7.5) at 45°C for 30 min and in 2×SSC for 2 min at room temperature. After dehydration in an ethanol series, DAPI (0.15 mg ml^−1^ in Vectashield Mounting Medium) was applied for chromatin counterstaining. Each FISH assay included one or two normal lung sections used as negative control, one or two sections from breast adenocarcinomas previously identified as carrying HER2 amplification used as positive control and five or six sections of lung carcinomas.

Microscopic analysis was performed on an Olympus BX60 brightfield and epifluorescence microscope equipped with the Quips XL genetic workstation (Applied Imaging, Santa Clara, CA, USA). Fluorescence signals were scored using single-band filters for DAPI, FITC, and Texas red, a double-band pass filter (FITC and Texas red) and a triple-band pass filter (DAPI, FITC and Texas red, Chroma Technology, Brattleboro, VT, USA). Representative images of each specimen were acquired with a SenSys cooled CCD camera (Photometrics, Tucson, AZ, USA) in monochromatic layers which were subsequently merged by the SmartCapture software (Vysis, Downers Grove, IL, USA).

The histological areas previously selected in the H&E-stained sections were identified in the FISH-treated slides. At least 200 non-overlapping interphase nuclei per site were scored for both HER-2/neu and chromosome 17 centromere signals, following strict scoring guidelines and constant adjustment of microscope focus since signals located at different focal planes. Two independent observers (L Chen and M Varella-Garcia) performed analysis in a blinded fashion. All requisite techniques involved were previously validated and results of scoring were found to be reproducible between the two independent operators.

### Prognostic implication of HER2 protein expression

Anonymous primary tumour tissue samples from patients diagnosed with non-small cell lung cancer pathological stage (pStage) I–III were obtained from the University of Colorado Cancer Center (UCCC) and the Johns Hopkins Medical Institutions (JHMI) from 1993 through 1999. The Colorado and the JHMI Institutional Review Boards approved the study protocol. A total of 187 patients with complete medical records were followed by the UCCC and JHMI tumour registries for survival time and outcome, and had adequate formalin-fixed, paraffin-embedded tissue blocks available for tissue microarray (TMA) construction. The study population included 111 males and 76 females with a median age of 65 years. Ninety-four per cent of the patients underwent thoracotomy, in which lobectomy were performed in the majority of cases. Surgical margins were positive for tumour involvement in 8%. Mediastinoscopy with lymph node resection was performed in the 11 non-resectable patients. The tumours were staged according to the International Union Against Cancer (UICC) TNM classification, and histologically subtyped and graded according to the WHO guidelines (Travis *et al*, 1999). Histopathologic examination revealed that 52% of the patients had pStage I disease. There were 95 SCC, 73 adenocarcinomas, 10 bronchoalveolar carcinomas (BAC), and 15 LCC. Of all tumours, 52% were poorly differentiated. Median follow-up was 51 months (range 18–100). Demographic and clinical data were collected retrospectively. None of the patients received radiotherapy or chemotherapy prior to surgery. The IHC procedure was the same as described above. All tumour and control tissues were reviewed by two pathologists (R Veve and WA Franklin).

### Tissue microarray construction

H&E stained sections were selected for tumour viability. Each slide was marked, and the point in the corresponding paraffin block was sampled for TMA construction. The TMAs were assembled using a tissue-arraying instrument (Beecher Instruments, Silver Springs, MD, USA), consisting of thin-walled stainless steel biopsy needles and stylets used to empty and transfer the needle content from the donor to recipient block. The assembly is held in an X–Y position guide that is manually adjusted by micrometers. Briefly, the instrument was used to create holes in a recipient paraffin block and to acquire tissue cores from the donor block by a thin-walled needle. The cylindrical sample was retrieved from the selected region in the donor block and extruded directly into the recipient block with defined array coordinates. A solid stylet, closely fit in the needle, was used to transfer the tissue cores into the recipient block. Taking tumour heterogeneity into account, we used a large diameter stylet (1.5 mm) and the study specimens were routinely over-sampled with three replicate core samples of tumour (different areas of the tumour) and normal (one, if present) regions, from each donor block. Normal lung and 15 other control specimens were included in each of the tissue array blocks. Multiple 4 μM sections were cut with a Leitz microtome. Sections were transferred to adhesive coated slides using the adhesive-coated tape sectioning system (Instrumedics, Hackensack, NJ, USA) as described by Mucci *et al* (2000). Subsequently, UV light treatment of the slides for 60 s polymerised the adhesive coating into a plastic layer and sealed the sections to the slides. The tape was then removed in a TPC solvent (Instrumedics, Hackensack, NJ, USA). The sections were then deparaffinized with standard xylene and hydrated through graded alcohols into water. One section from each tissue array block was H&E stained and coverslipped. The remaining sections were stored at room temperature for IHC staining.

### Statistical analysis

Spearman correlation coefficient was used to examine the relationship between the maximum and the average number of the HER-2/neu gene per cell and the ratio gene/chromosome by histology. The Mantel–Haenszel Chi-square test was applied to examine the differences in linear trend of HercepTest score and HER-2/neu copy number per cell across the histologic subtypes of lung cancer. For the survival analysis, the Kaplan–Meier method was applied to estimate the lung cancer-related survival for each category of HercepTest score. The log-rank test was applied to examine the association between survival and HercepTest score. All statistical analyses were carried out with SAS software (SAS Institute, Cary, NC, USA).

## RESULTS

Altogether, 238 patients with various NSCLC histologies were included in the analyses. Thirty-nine patients (16%) had positive HercepTest score (2+/3+), but only nine patients (4%) had a strong positive result (3+). The distribution of the results from the HercepTest according to the histological types is shown in Table 1

**Table 1:**
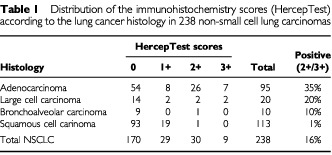
Distribution of the immunohistochemistry scores (HercepTest) according to the lung cancer histology in 238 non-small cell lung carcinomas

**Figure 1:**
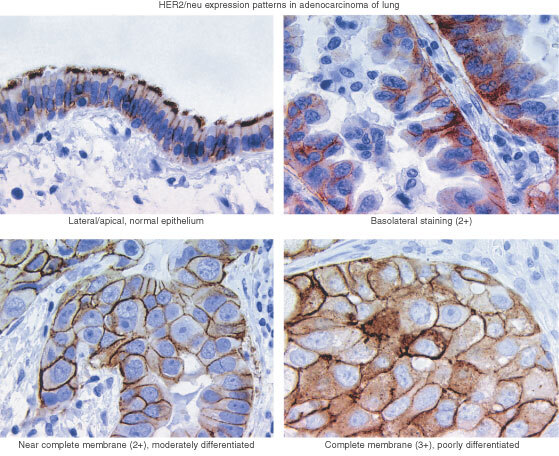
Typical staining patterns by immunohistochemistry (HercepTest, Dako, Denmark): Normal epithelium with apical/lateral staining, but no basocellular staining (*Upper left*). Modified 2+with a strong basolateral staining seen in some adenocarcinomas, but without complete membrane staining (*Upper right*). A moderate near complete membrane staining in more than 10% of the cells, considered as 2+(*lower left*). Complete strong membrane staining, considered as 3+(*lower right*).

. Among 95 patients with adenocarcinomas, 33 patients (35%) had a HercepTest positive tumour, and four out of 20 LCC patients (20%) had a positive tumour. Among 113 SCC patients, only one (1%) had a HercepTest positive tumour. Typical staining patterns are illustrated in Figure 1

**Figure 3:**
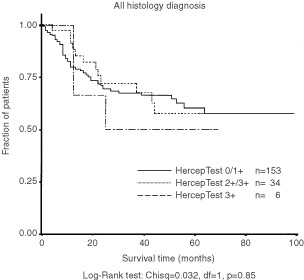
Kaplan–Meier survival curves according to the results of HercepTest.

.

### Correlation between FISH and IHC

Tumours from 51 of the patients were evaluated by both IHC and FISH techniques. The distribution of the IHC results in this subset was not statistically different from the total set of 238 patients. Strong positive staining (3+) was observed in 6% of tumours, a positive 2+ result was found in 19%, and 75% of the tumours were negative (0–1+). The highest rates of positive staining were found among the adenocarcinomas (42%) and LCC (40%), while only 5% of the SCC were positive. The difference across histological subtypes was statistically significant (*P*<0.05, Mantel–Haenszel Chi-square test for linear association).

In the FISH study each tumour was assessed by the average and the maximum numbers of copies of the HER-2/neu gene per cell, the average ratio of HER-2/neu gene to chromosome 17 copy numbers, and the pattern of signal presentation (Tables 2

**Table 2:**
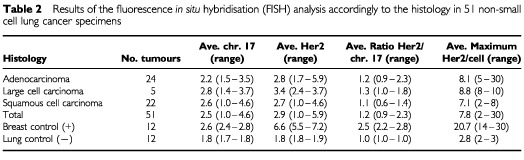
Results of the fluorescence *in situ* hybridisation (FISH) analysis accordingly to the histology in 51 non-small cell lung cancer specimens

and 3

**Table 3:**
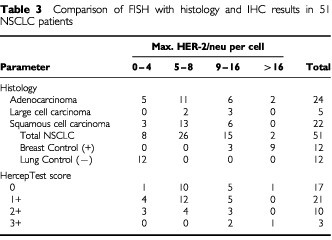
Comparison of FISH with histology and IHC results in 51 NSCLC patients

, Figure 2

**Figure 2:**
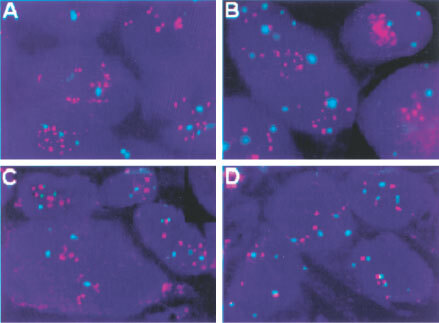
Dual-colour FISH assay using the PathVysion DNA probe (Vysis) in lung tissue sections. HER-2/neu probe is labelled in red (SpectrumOrange) and chromosome 17 probe is labelled in green (SpectrumGreen). Chromatin in staining in blue (DAPI). Gene amplification (Gene/Chromosome ratio >2.0) is illustrated in patients with adenocarcinomas with HercepTest score 0 in (**A**) and HercepTest score 3+ in (**B**). Small HER-2/neu clusters (Gene/Chromosome ratio 1.2) are illustrated in (**C**) for a patient with squamous cell carcinoma and HercepTest 1+. Balanced aneusomy (Gene/Chromosome ratio 1.0) is shown in an adenocarcinoma patient with HercepTest score 2+ (**D**).

). There was a strong and significant correlation between maximum and average HER-2/neu copy numbers per cell (Spearman correlation coefficient=0.93; *P*<0.001). There was also a significant correlation between the maximum HER-2/neu copy number and the ratio of HER-2/neu to chromosome 17 (Spearman correlation coefficient=0.54, *P*<0.001).

The average number of HER-2/neu gene signals per cell was 2.9 and the average number of chromosome 17 signals was 2.6. The average ratio gene/chromosome signals was 1.2. Of these 51 NSCLCs, only two (4%) had a maximum of >16 copies of the HER-2/neu gene per cell (mean=five copies/cell) with a gene/chromosome ratio >2, indicating true gene amplification. The HER-2/neu signals in these two tumours were presented in a clustered pattern, consistent with that expected in homogenously staining regions (Figure 2A,B). Fifteen cases (29%) had a maximum of 9–16 copies of the gene per cell (mean=three copies/cell). Small clusters of signals were observed in seven of these 15 samples (Figure 2C). In 26 cases (55%), the maximum number of copies per cells was 5–8 (mean=2.6 copies/cell), and in eight cases (16%) was <5 (mean=1.6 copes/cell). Except for the two tumours with clustered amplification, all other tumours had gene/chromosome ratios ⩽1.5. Therefore, in the NSCLC tumours, increased number of copies of HER-2/neu per cell were largely due to chromosomal polysomy and both fluorescent signals (red for the gene, green for the chromosome 17) displayed a scattered pattern as illustrated in Figure 2D.

In the normal lung specimens used as negative controls, the maximum number of copies of the HER-2/neu gene per cell was three, the average was 1.8 and the average ratio gene to chromosome was 1.0. In contrast, all of the 12 breast cancer samples used as positive controls had a clustered HER-2/neu gene amplification. The maximum number of copies ranged from 14 to 30 and averaged 21.0. The average number of copies per cell was 6.6 and the average ratio of gene/chromosome was 2.5.

The HER-2/neu gene copy number per cell did not differ significantly among the NSCLC histologic subtypes (*P*>0.05 in the Mantel–Haenszel Chi-Square test), but the normal lung samples were statistically different from the cancer samples. The maximum number of copies of HER-2/neu per cell averaged 8.1 in adenocarcinomas, 8.8 in LCC, and 7.1 in SCC. The average copy number of HER-2/neu per cell was 2.8 in adenocarcinomas, 3.4 in LCC, and 2.7 in SCC. As presented in Table 3, both of the lung tumour samples with a maximum number of HER-2/neu copies per cell >16 were adenocarcinomas (4%). Among the 15 lung cancer samples with a maximum of 9–16 copies of HER-2/neu per cell, six were adenocarcinomas, six were SCC and three were LCC. Among the 34 tumours with a maximum <9 copies of HER-2/neu per cell, there were 16 adenocarcinomas, 16 SCC, and two LCC.

Twenty-seven tumours (53%) with HercepTest score of 0/1+ and a maximum number of copies of HER-2/neu per cell from <9 were considered negative by both analyses. Six tumours (12%) with HercepTest score 2+/3+ and the maximum number of copies of HER-2/neu per cell ⩾9 were considered positive by both analyses. Discordant results were observed in 18 cases (35%). In seven (14%), the HercepTest was 2+ and the maximum number of copies of HER-2/neu per cell was <9; in the remaining 11 (21%) there was a maximum of ⩾9 copies of HER-2/neu per cell and the HercepTest score was 0 or 1+.

### Prognostic impact of HER-2/neu protein expression

Altogether 187 patients with NSCLC were examined for prognosis related to HER-2/neu expression. Thirty-four tumours (18%) were positive by HercepTest (2+/3+), but only six (3%) were strongly positive (3+). Among 91 SCC, 8 tumours (9%) were positive and all of them had 2+ HercepTest score. In contrast, among the 96 tumours with non-squamous histologies, 26 (27%) had a positive HercepTest score, and six of them were 3+. Seventy-one tumours were adenocarcinomas, from which 23 (32%) were positive and five (7%) were 3+.

Kaplan–Meier survival curves for the different groups are presented in Figure 3. Based on the 187 patients, no difference in survival was found comparing patients with positive (HercepTest 2+/3+, *n*=34) and negative (HercepTest 0/1+, *n*=153 pts) tumours (*P*=0.86). The 3-year/5-year survival was 70/58% for the HER-2/neu positive patients, and 66/60% for the negative patients. When the 3+ patients were analysed separately (*n*=6), the survival for these patients tended to be shorter (3-year/5-year survival=50%). However, statistical analysis was not performed due to the small number of patients. No difference in survival was observed for patients with squamous histology comparing those with HER-2/neu positive (*n*=8) and negative (*n*=83) tumours (*P*=0.60). Similarly, no difference in survival was observed comparing patients with positive (*n*=26) and negative (*n*=70) tumors in the non-squamous histology categories (*P*=0.91).

## DISCUSSION

In the current study, we found HER-2/neu overexpression (2+ and 3+) by IHC in 16% of the NSCLC tumours, most frequently in adenocarcinomas (35%) and LCC (20%). These results are similar to most reports of NSCLC in the literature (Schneider *et al*, 1989; Kern *et al*, 1990; Tateishi *et al*, 1991; Paakko *et al*, 1992; Tsai *et al*, 1993; Hsieh *et al*, 1998). Exceptions are some recent studies (Cox *et al*, 2001; Hirashima *et al*, 2001), in which lower frequencies of NSCLC tumours with HER-2/neu overexpression were reported. Discrepancies among the reported frequencies might be due to different factors, including distribution of histological types, tissue processing, criteria for the scoring and interpretation of the staining results.

No standard immunohistochemical protocol has been developed for lung cancer. The HercepTest was developed and approved by the US FDA for evaluation of HER-2/neu in breast cancer. Lung tumours are, however, morphologically more heterogeneous than malignant breast tumours, which increases the difficulties in the interpretation of the HER-2/neu staining results. We observed that a few lung adenocarcinomas (<5%) had a strong basolateral staining, but not a complete membrane staining. Because of the incomplete membrane staining, these tumours might have been classified by others as ‘negative’, according to the HercepTest. However, because of the strong intensity of the staining pattern, we classified these tumours as having 2+ results (Figure 1). This could give a slightly higher frequency of positive adenocarcinomas compared to other studies (Cox *et al*, 2001; Hirashima *et al*, 2001). However, the clinical significance of this scoring may not be relevant because 2+ tumours do not have a worse prognosis and may not have a significant benefit from HER-2/neu targeted therapy.

The HER-2/neu gene status has been scarcely investigated in lung tumours. We evaluated the copy number of HER-2/neu sequences per cell using a dual-colour FISH assay which included the gene probe and the centromere probe as a control. There is no uniform criterion to determine gene amplification by FISH. There is a general consensus that gene/chromosome ratio >2 represents gene amplification, however, other parameters have also been used. For instance, Pauletti *et al* (2000) found that the maximum number of copies of HER-2/neu in breast cancer cells correlated with the worsening of the prognosis supporting that this is a good index for assessing amplification. In the current study, we demonstrated that the maximum number of copies of HER-2/neu per cell in lung tumours was positively correlated both with the average number of HER-2/neu per cell and the gene/chromosome ratio. Therefore, either one of these indices may be useful for studying prognostic and therapeutical implications of the HER-2 overexpression.

When comparing IHC and FISH results, the HercepTest showed positive results (2+/3+) in 25% of cases while the FISH showed ⩾9 copies of the HER-2/neu gene per cell in 32% of cases but ⩾16 copies of HER-2/neu per cell in only 4% of cases. Overall, HER-2/neu overexpression was more common in adenocarcinomas and LCC and less common in SCC both at the surface protein level and at the gene level.

HER-2/neu gene amplification, represented by the gene/chromosome ratio >2 was uncommon in the NSCLCs (two patients=4%) and even when considering the adenocarcinomas, the fraction of tumours with a maximum HER-2/neu copy number per cell >16 was 8%. Other studies, such as Hirashima *et al* (2001) and Cox *et al* (2001) also detected clustered amplification only in rare cases of NSCLC. Disomic status for chromosome 17 was only found in 16% of the NSCLC tumours included in our study. In the majority of these tumours, there were increased numbers of copies of HER-2/neu gene per cell accompanied by a balanced chromosome gain (gene/chromosome ration ∼1). Since low levels of HER-2/neu cell surface protein expression was the most common finding in these tumours, the results support the conclusion that the extensive chromosome 17 polysomy detected in lung tumours is not associated with HER-2/neu protein overexpression.

Other detailed studies comparing the levels of HER-2/neu protein expression by IHC with different levels of gene gain/amplification by FISH have not been published in lung tumours. Comparing our results with the large series of breast tumours reported by Pauletti *et al* (2000), a marked gene amplification and protein overexpression was considerably more common in breast than in lung cancers (13% *vs* 4% for FISH results, 11% *vs* 6% for IHC results). Similarly, the complete lack of protein surface expression (score 0) and gene copy numbers per cell <4 were also more common in breast than in lung cancer (55% *vs* 15% for FISH, 70% *vs* 36% for IHC). None of the lung cancers in our series, including adenocarcinomas, had a maximum copy number per cell >32 whereas 5% of the breast cancers have been reported in this category (Pauletti *et al*, 2000). More often lung cancers had marked chromosomal aneusomy, with a balanced increase in the number of copies of chromosome 17 and HER-2/neu gene copies. This balanced increase does not represent true gene amplification and its clinical implications remain to be determined.

There was a good correlation between the IHC and FISH results in two-thirds of the tumours investigated. In the discrepant cases, there were either increased aneusomy (no gene amplification) without increased levels in the protein expression or increased protein expression without significant increase in the number of copies of the gene. Discrepancies between protein expression (IHC) and the gene status (FISH) have also been reported in breast tumours (Tubbs *et al*, 2001). Interestingly, one of our patients with true gene amplification by FISH had a negative score by IHC (Figure 2A). There are several potential reasons for these discrepant results comparing IHC with FISH. The discrepancies might be due to quality of fixation and tissue processing, as well as disturbances in the transcriptional or post-transcriptional controlling mechanisms.

The clinical relevance of gene amplification or protein overexpression of HER-2/neu in HSCLC as detected by FISH and IHC assays remains to be determined. Applying the HercepTest to a tissue microarray of 187 surgically resected lung tumours, we could not demonstrate any statistically significant difference in prognosis between patients with negative (0/1+) and with positive tumours (2+/3+). A tendency of shorter survival was observed for patients with 3+ tumours but no statistical analysis could be done due to the small number of patients in this category.

Theoretically it might be predicted that the protein expression would be superior for assessing response to trastuzumab therapy because the antibody binds to the cell surface protein. In breast cancer, however, FISH technique has been demonstrated to be a more accurate and reliable method for selecting patients eligible for treatment with trastuzumab (Jacobs *et al*, 1999; Mass *et al*, 2000; Pauletti *et al*, 2000; Tubbs *et al*, 2001). In our previous studies with lung cancer cell lines, we detected a positive correlation between HER-2/neu expression by IHC and effectiveness of treatment with trastuzumab alone or in combination with cytostatic drugs (Bunn *et al*, 2001). In lung cancer patients, we currently do not know which level of HER-2/neu protein expression or gene gain/amplification will have prognostic/therapeutic implications. These questions need to be addressed in prospective trials. Based on the clinical experience from the breast cancer studies, it might be of interest to focus on NSCLC patients with 3+ IHC results and/or those having tumours with gene amplification. Data from the current and other studies (Cox *et al*, 2001) show that these conditions seem to be limited to adenocarcinomas and LCC. Thus, the number of patients who might be candidates for trastuzumab treatment seems to be more limited in the NSCLC population than in the breast cancer population, and such prospective clinical trials in NSCLC will require multicenter studies. However, taken the large amount of NSCLC patients into account, still many patients might benefit from such a treatment. Clinical trials with trastuzumab in lung cancer should assess HER-2/neu expression by both methods to determine which test is superior for predicting response.
